# The Effect of Precipitation on the Transmission of Japanese Encephalitis (JE) Virus in Nature: A Complex Effect on Antibody-Positive Rate to JE Virus in Sentinel Pigs

**DOI:** 10.3390/ijerph10051831

**Published:** 2013-05-03

**Authors:** Ichiro Kurane, Ken-ichi Shibasaki, Akira Kotaki, Yasuaki Hijioka, Tomohiko Takasaki

**Affiliations:** 1 National Institute of Infectious Diseases, 1-23-1 Toyama, Shinjukuku, Tokyo 162-8640, Japan; 2 Department of Virology 1, National Institute of Infectious Diseases, 1-23-1 Toyama, Shinjukuku, Tokyo 162-8640, Japan; E-Mails: shi-k@pcpod.jpn.org (K.S.); ak@nih.go.jp (A.K.); takasaki@nih.go.jp (T.T.); 3 Sustainable Social Systems Section, Center for Social and Environmental Systems Research, National Institute for Environmental Studies, 16-2 Onogawa, Tsukuba-City, Ibaraki 305-8506, Japan; E-Mail: hijioka@nies.go.jp

**Keywords:** Japanese encephalitis, pigs, climate change, precipitation

## Abstract

Japanese encephalitis (JE) is one of the most important mosquito-borne viral diseases in Asia. Pigs are a natural host and the amplifier of JE virus. The sero-conversion rate to JE virus in sentinel pigs reflects the activity of JE virus in the region. We analyzed whether precipitation has any effect on the sero-conversion rate to JE virus in sentinel pigs. Linear regression analysis was performed to determine the correlations between the levels of precipitation and sero-conversion rates to JE virus, in the entire year and during summertime over the period of 32 years from 1969 to 2000. The levels of the annual and summertime precipitation demonstrated statistically significant positive correlations with sero-conversion rates for the whole of the country and for some regions in Japan. The levels of the summertime precipitation, on the other hand, demonstrated statistically significant inverse correlations with the sero-conversion rates in other regions. Further, the levels of precipitation during preceding 10-day periods from days 1–40 before blood collection showed inverse correlation with antibody-positive rates in some regions. The results indicate that the relationship between the annual and summertime precipitation, and the sero-conversion rate to JE virus is complex; both positive and inverse effects are demonstrated depending on the regions.

## 1. Introduction

Climate change affects various aspects of human health, including infectious diseases. Vector-borne infectious diseases and water-borne infectious diseases are two main categories of infectious diseases that are forecasted to be most affected [1,2,3]. However, the levels of the effects and the mechanisms have not been fully understood [4,5,6]. The levels and types of the effects on the same infectious disease may be different, depending on the geographical location and the socio-economic status.

Japanese encephalitis (JE) is a mosquito-borne viral encephalitis with a high mortality rate and a high percentage of neuro-psychiatric sequelae [7]. JE occurs endemically and/or epidemically in many Asian countries. Approximately 50,000 cases of JE have been reported annually worldwide [7]. The principal vector of JE virus in East Asia, including Japan, is *Culex tritaeniorhynchus*. JE virus is maintained in Nature between vector mosquitoes and domestic pigs in endemic regions [8]. Pigs also act as the amplifier for JE virus [8]. Naïve pigs are highly susceptible to JE virus. They develop high levels of viremia and specific antibody after infection with JE virus by bite of infected mosquitoes. Uninfected mosquitoes are then infected by biting infected pigs with high levels of viremia. 

The number of human JE cases is determined by multiple factors such as the activity and numbers of vector mosquitoes, the location of pig farms, and the percentage of protective antibody-positivity in humans. The levels of JE virus activity in nature, thus, is not directly reflected by the number of JE cases in the countries where JE vaccination has been strongly implemented and most of the population have protective immunity. In those countries, the most reliable parameter of JE virus activity is sero-conversion rate among sentinel naive pigs [8,9]. 

The vector mosquitoes begin to be detected in May, sero-conversion of pigs begins and occurrence of human cases follows in Japan [9]. Pigs are usually slaughtered to be shipped to the market before 6 months of age. Most pigs are, thus, born after JE season is over in the previous year. The newly born pigs are naïve to JE virus when the new epidemic season starts in the following year. 

We have reported that temperature has positive effect on sero-conversion rates to JE virus in sentinel pigs (Kurane *et al.*, submitted manuscript). The effect of precipitation on the sero-conversion rates among pigs has not been well understood. In the present study, we analyzed whether annual and summertime precipitation has any effect on the sero-conversion rates among sentinel pigs in Japan. There are positive correlations between the precipitation and sero-conversion rates for the whole of Japan. On the other hand, there are inverse correlations in some of the regions. The results suggest that the effects of precipitation on the sero-conversion rates in sentinel pigs are complex, and that the patterns of rainfalls may determine the effect.

## 2. Experimental Section

### 2.1. Sero-Conversion rate in Sentinel Pigs

JE surveillance has been implemented since 1965 in the National Epidemiological Surveillance of Vaccine Preventable Diseases by the Ministry of Health and Welfare (currently the Ministry of Health, Labour and Welfare) of Japan [9]. Sero-conversion rates to JE virus in sentinel pigs were assessed by measuring hemagglutination inhibition (HI) antibody to JE virus 2–3 times a month by the member of vaccine preventable diseases surveillance program of Japan from May to October. Sero-conversion rates of sentinel pigs from 1969 to 2000 in the prefectures throughout Japan were obtained from the Reports of the National Epidemiological Surveillance of Vaccine Preventable Diseases by the Ministry of Health, Labour and Welfare, and used in the present study. Two types of data were used as the sero-convertion rate; the mean HI anibody-positive rate and the maximum HI antibody-positive rate during the survey period in respective years in each of the participating prefectures. These two parameters were used, because they represent different aspects of JE virus activities in Nature.

**Figure 1:**
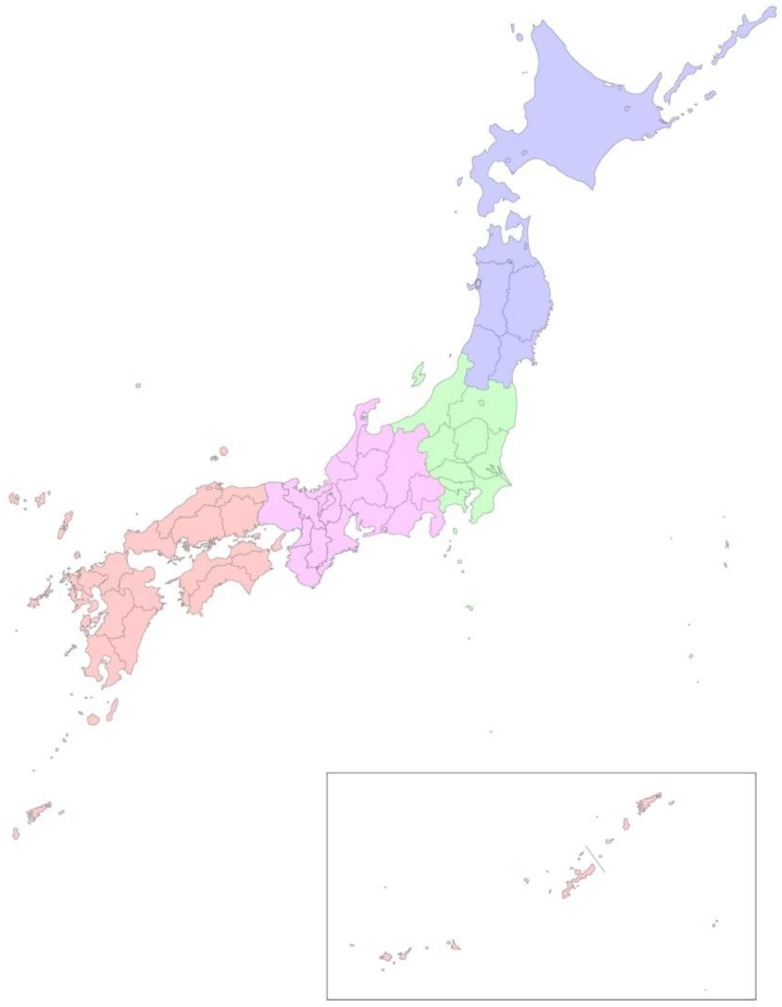
Four regions of Japan designated in the study.Japan was divided into four regions according to the locations: north (purple), central (green), west (pink) and south (orange).

### 2.2. Meteorological Data

The annual precipitation from 1969 to 2000 in the respective cities was obtained from the Japan Meteorological Agency [9]. The meteorological data of the cities where the pig farms or slaughter houses were located were used. When the meteorological data were not available at the cities, the data of the closest cities available within the respective prefectures were used. The annual means of the daily precipitation and the means during summertime (June, July and August) for each prefecture were calculated and used for the analyses. 

### 2.3. Statistical Analyses

Linear regression analysis was performed to determine the correlation between antibody-positive rate in sentinel pigs, and the annual precipitation and that during summertime (June, July and August), for each of the participating prefectures. In some analyses, Japan was divided into four regions: region 1 (north), region 2 (central), region 3 (west) and region 4 (south), as indicated in Figure 1. It takes several weeks for newly hatched mosquitoes to be able to transmit JE virus. The effects of the rainfall during preceding 10 day-periods from days 1–50 before blood collection on sero-conversion rates were, therefore, also analyzed. R^2^ is the coefficient of determination. *P* values lower than 0.05 were defined to be statistically significant.

## 3. Results and Discussion

### 3.1. The Relationship between the Annual Precipitation and Sero-Conversion Rate in Sentinel Pigs

The relationship was analyzed between the annual precipitation and the levels of antibody-positive rates. Two types of the sero-conversion data were used: the mean and the maximum sero-conversion rates in respective years. The relationships were analyzed for the whole of Japan, and for each of the four regions shown in Figure 1 (Table 1). 

**Table 1:** Relationship between the annual and summertime precipitation and sero-conversion rate to JE virus in sentinel pigs.

Periods and Regions	Sample Number	Mean HI positivity			Maximum HI positivity		
		Inclination	R^2^	*p*	Inclination	R^2^	*p*
Year-round							
Entire Japan	947	0.0096	0.0446	1.899 × 10^−11^	0.0153	0.0529	7.790 × 10^−13^
Region 1 (North)	167	0.0070	0.0223	0.054	0.0188	0.0250	0.041
Region 2 (Central)	280	0.0031	0.0069	0.166	0.0053	0.0065	0.179
Region 3 (West)	199	−0.0061	0.0154	0.081	−0.0094	0.0399	0.005
Region 4 (South)	301	0.0045	0.0183	0.019	0.0027	0.0063	0.170
June, July and August							
Entire Japan	941	0.0474	0.0387	1.156 × 10^−9^	0.0706	0.0384	1.344 × 10^−9^
Region1 (North)	165	0.0009	0.00001	0.968	0.0229	0.0012	0.659
Region 2 (Central)	279	−0.0468	0.0174	0.028	−0.0942	0.0225	0.012
Region 3 (West)	197	−0.0677	0.0574	0.001	−0.0850	0.0969	8.490 × 10^−6^
Region 4 (South)	300	0.0137	0.0078	0.127	0.0061	0.0014	0.519

**Figure 2:**
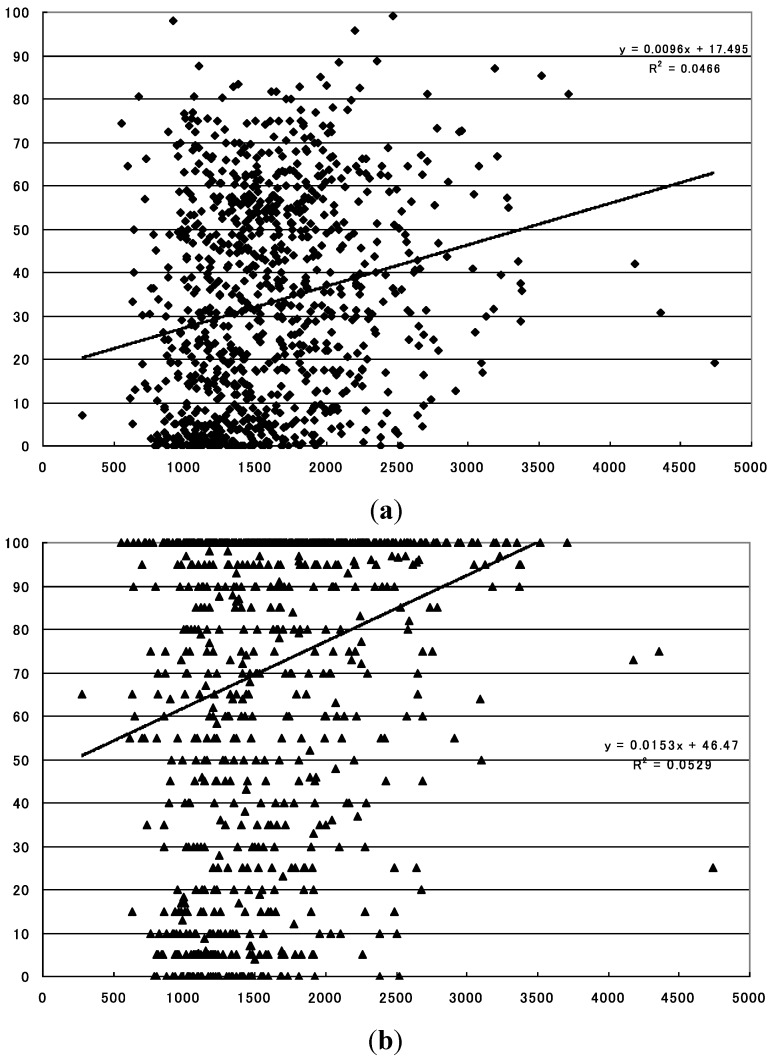
Relationship between annual precipitation and sero-conversion rate to JE virus in sentinel pigs in the whole of Japan. (**a**) Statistically significant positive relationship with the mean sero-conversion rate (*p* = 1.899 × 10^−11^).X-axis: precipitation during the period (mm), Y-axis: sero-conversion rate to JE virus (%); (**b**) Statistically significant positive relationship with the maximum sero-conversion rate (*p* = 7.790 × 10^−13^). X-axis: precipitation during the period (mm), Y-axis: sero-conversion rate to JE virus (%).

The levels of the annual precipitation demonstrated statistically significant positive correlations with both the mean and maximum sero-conversion rates for the whole of the country (*p* = 1.899 × 10^−11^ with the mean sero-conversion rate, and *p* = 7.790 × 10^−13^ with the maximum sero-conversion rate) (Figure 2). The levels of annual precipitation also demonstrated statistically significant positive correlations with the mean sero-conversion rates for region 4 (*p* = 0.019), and with the maximum sero-conversion rate for region 1 (*p* = 0.041) (Table 1). On the other hand, the levels of the annual precipitation demonstrated statistically significant inverse correlations with the maximum sero-conversion rate for region 3 (*p* = 0.005).

### 3.2. The Relationship between the Summertime Precipitation and Sero-Conversion Rate in Sentinel Pigs

**Figure 3:**
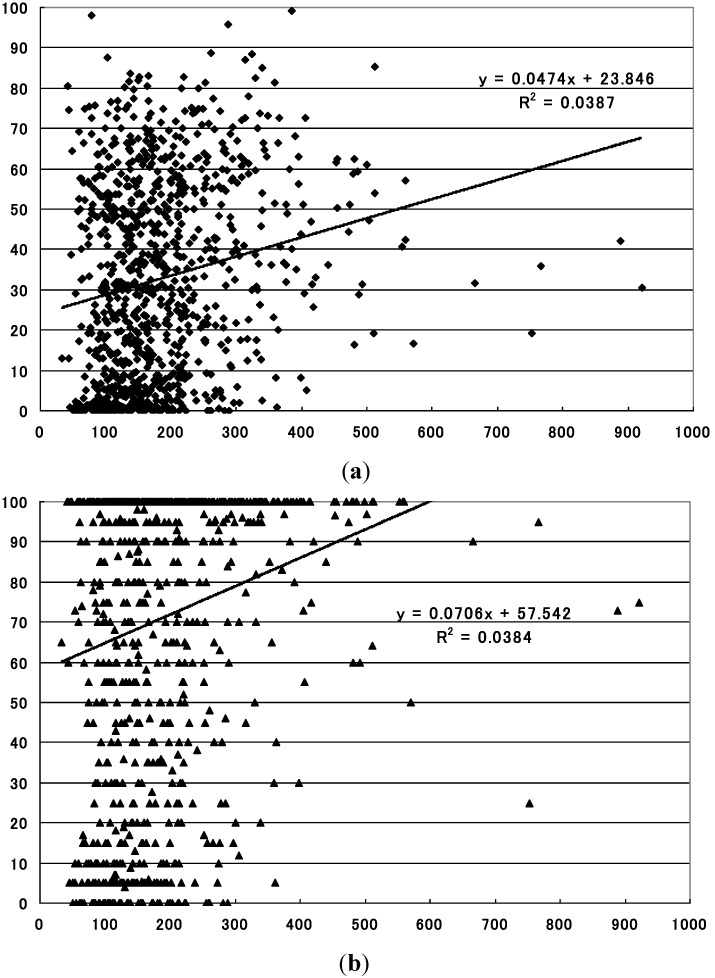
Relationship between summertime precipitation and sero-conversion rate to JE virus in sentinel pigs in the whole of Japan. (**a**) Statistically significant positive relationship with the mean sero-conversion rate (*p* = 1.156 × 10^−9^). X-axis: precipitation during the period (mm), Y-axis: sero-conversion rate to JE virus (%); (**b**) Statistically significant positive relationship with the maximum sero-conversion rate (*p* = 1.344 × 10^−9^). X-axis: precipitation during the period (mm), Y-axis: sero-conversion rate to JE virus (%).

The levels of the summertime precipitation demonstrated statistically significant positive correlations with both the mean and maximum sero-conversion rates for the whole of Japan (*p* = 1.156 × 10^−9^ with the mean sero-conversion rate, and *p* = 1.344 × 10^−9^ with the maximum sero-conversion rate) (Table 1, Figure 3). 

**Figure 4:**
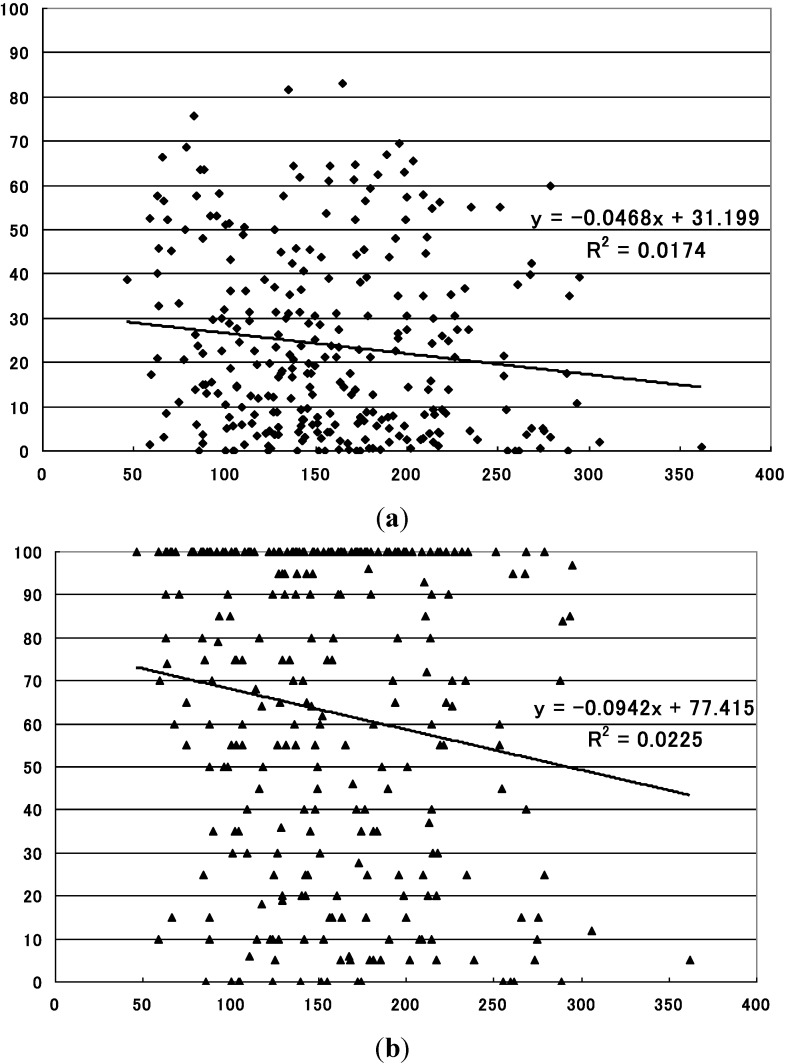
Relationship between summertime precipitation and sero-conversion rate to JE virus in sentinel pigs in the region 2 (central region). (**a**) Statistically significant inverse relationship with the mean sero-conversion rate (*p* = 0.028). X-axis: precipitation during the period (mm), Y-axis: sero-conversion rate to JE virus (%); (**b**) Statistically significant inverse relationship with the maximum sero-conversion rate (*p* = 0.012). X-axis: precipitation during the period (mm), Y-axis: sero-conversion rate to JE virus (%).

**Figure 5:**
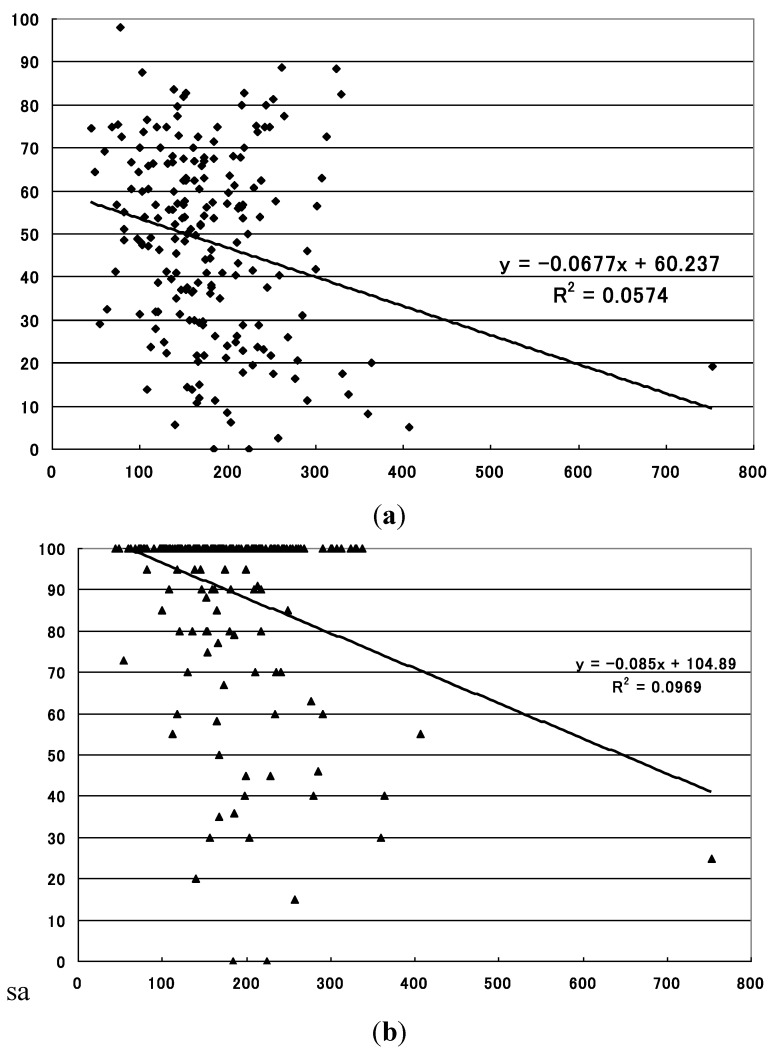
Relationship between summertime precipitation and sero-conversion rate to JE virus in sentinel pigs in the region 3 (west region). (**a**) Statistically significant inverse relationship with the mean sero-conversion rate (*p* = 0.001). X-axis: precipitation during the period (mm), Y-axis: sero-conversion rate to JE virus (%); (**b**) Statistically significant inverse relationship with the maximum sero-conversion rate (*p* = 8.490 × 10^−6^). X-axis: precipitation during the period (mm), Y-axis: sero-conversion rate to JE virus (%).

The levels of the summertime precipitation, on the other hand, demonstrated statistically significant inverse correlations with the mean sero-conversion rates for region 2 (*p* = 0.028) and region 3 (*p* = 0.001), and with the maximum sero-conversion rate for region 2 (*p* = 0.012) and for region 3 (*p* = 8.490 × 10^−6^) (Table 1, Figure 4, Figure 5).

### 3.3. The Effect of the Precipitation during Previous Days on the Sero-Conversion Rate to JE Virus in Pigs

We determined whether the levels of precipitation during preceding days before blood collection have any effect on the sero-conversion rate. The relationship was analyzed between the maximum sero-conversion rate in sentinel pigs and the levels of rainfall during preceding 10-day periods from days 1–50 before blood collection (Table 2).

**Table 2:** Effect of the precipitation during preceding 10-day periods from day 1 to day 50 before blood collection on the maximum sero-conversion rate to JE virus in sentinel pigs.

Period	Sample Number	Inclination	R^2^	*p*
1–10 days				
Entire Japan	6,580	−0.2876	0.0026	3.502 × 10^−5^
Region1 (North)	879	0.5632	0.0148	3.002 × 10^−4^
Region 2 (Central)	2,078	0.1385	0.0006	0.264
Region 3 (West)	1,599	−0.9662	0.0191	2.878 × 10^−8^
Region 4 (South)	2,024	−0.6188	0.0191	4.273 × 10^−10^
11–20 days				
Entire Japan	6,590	−0.4262	0.0059	4.279 × 10^−10^
Region1 (North)	882	0.4695	0.0110	0.002
Region 2 (Central)	2,080	−0.3444	0.0033	0.009
Region 3 (West)	1,599	−1.4403	0.0409	3.210 × 10^−16^
Region 4 (South)	2,029	−0.5990	0.0188	5.586 × 10^−10^
21–30 days				
Entire Japan	6,593	−0.3330	0.0037	7.706 × 10^−7^
Region1 (North)	886	0.2694	0.0031	0.098
Region 2 (Central)	2,079	−0.7207	0.0142	5.050 × 10^−8^
Region 3 (West)	1,598	−1.4713	0.0414	2.150 × 10^−16^
Region 4 (South)	2,030	−0.3450	0.0068	1.997 × 10^−4^
31–40 days				
Entire Japan	6,588	−0.0570	0.0001	0.417
Region1 (North)	883	0.3161	0.0038	0.067
Region 2 (Central)	2,069	−0.3561	0.0031	0.011
Region 3 (West)	1,605	−1.0472	0.0210	5.480 × 10^−9^
Region 4 (South)	2,031	−0.1814	0.0017	0.063
41–50 days				
Entire Japan	6,596	0.4577	0.0063	1.080 × 10^−10^
Region1 (North)	885	0.2640	0.0023	0.154
Region 2 (Central)	2,090	−0.0575	0.0001	0.683
Region 3 (West)	1,604	−0.2592	0.0012	0.166
Region 4 (South)	2,017	0.3596	0.0065	2.895 × 10^−4^

The levels of precipitation during preceding days demonstrated statistically significant inverse correlations with sero-conversion rates during preceding days 1–10 for the whole of Japan (*p* = 3.502 × 10^−5^), region 3 (*p* = 2.878 × 10^−8^) and region 4 (*p* = 4.273 × 10^−1^^0^), during preceding days 11–20 for the whole of Japan (*p* = 4.279 × 10^−1^^0^), region 2 (*p* = 0.009), region 3 (*p* = 3.210 × 10^−16^) and region 4 (*p* = 5.586 × 10^−1^^0^), during preceding days 21–30 for the whole of Japan (*p* = 7.706 × 10^−7^), region 2 (*p* = 5.050 × 10^−8^), region 3 (*p* = 2.150 × 10^−16^) and region 4 (*p* = 1.997 × 10^−4^), and during preceding days 31–40 for region 2 (*p* = 0.011) and region 3 (*p* = 5.480 × 10^−9^). On the other hand, the levels of precipitation demonstrated statistically significant positive correlations with sero-conversion rates during preceding days 1–10 for region 1 (*p* = 3.002 × 10^−4^), during preceding days 11–20 for region 1 (*p* = 0.002), and during preceding days 41–50 for region 4 (*p* = 2.895 × 10^−4^).

### 3.4. Discussion

The effect of precipitation on the sero-conversion rate to JE virus in sentinel pigs was examined. As the meteorological parameter, we used: (i) the annual precipitation; (ii) the precipitation during summertime (June, July and August). The data on percent antibody-positive rates were used from 1969 to 2000, because high levels of sero-conversion to JE virus were constantly detected during this period. The results suggest that the relationship between the annual and summertime precipitation, and the sero-conversion rate to JE virus is complex; there were statistically significant positive correlations in the whole of country and in some of the regions; and there were also statistically significant inverse correlation in other regions. 

Pigs are a natural host and the amplifier of JE virus in the JE virus transmission cycle in nature [7]. In Japan, the vector mosquitoes begin to be detected in May [9]. Sero-conversion of sentinel pigs then occur and the occurrence of human JE cases follows. The principal vector of JE virus in east Asia, including Japan, is *Culex tritaeniorhynchus* [8,10], which reproduces in rice paddies and the connecting canals. It prefers the blood of middle to large-sized domestic or wild animals and that of humans. It is active in twilight and bites outdoors. It was reported that there was a positive relationship between the number of mosquitoes and that of JE cases [11]. The complex effect of precipitation on the antibody-positive rate in pigs may be in part due to positive and negative effects of precipitation on life-cycle of the vector mosquito. Certain levels of precipitation are needed to maintain water enough for survival of larvae. High levels of rainfalls for short period of time may washout the larvae, thus, decrease the number of mosquitoes in the area.

The life cycle of *Culex tritaeniorhynchus* includes 2 days of egg period, 8–10 days of larva period and 1–2 days of pupa period, making a total of 11–14 days. Furthermore, it takes 10–12 days for the mosquito which bit the infected pigs to become able to transmit the virus to naïve pigs. It will also take 5–7 days for the infected pig develop specific antibody to JE virus. The calculation, on one hand, makes the results reasonable that the levels of rainfall during previous 10–40 days may wash out larvae, and demonstrate inverse correlation with antibody-positive rate in some regions. It is, however, difficult to explain positive correlations in other regions. The pattern of rainfalls may have undefined effects on the antibody-positive rates to JEV. Further studies are needed to determine the discrepancies of the results.

It has been reported that climate change has effects on arboviral diseases [1,2,3]. There have been reports on the effect of climate change on JE [12,13]. In those papers, the number of JE cases was used for analyses. The number of JE patients is affected by multiple factors, such as antibody-positive rate in the population, levels of vaccine implementation, distributions of rice paddies and pig farms, life-style and social infrastructure, and JE virus virulence, as well as climate changes. Even though the level of the activity of JE virus-infected mosquitoes has increased, the number of JE patients has not increased in the countries where most of the populations have protective immunity against JE virus. Thus, the number of JE cases may not directly reflect the activity of JE virus in the countries, including Japan, where JE vaccination has been widely implemented. Pigs are slaughtered to be shipped to the market before 6 months of age, and the pigs present in May and following months were born after the JE season ended in previous year. They are naïve to JE virus when the new epidemic season starts in the next year. Naïve pigs are susceptible to JE virus, and develop high levels of viremia and specific antibody after infection with JE virus. Pigs are not immunized with JE vaccine Japan. Sero-conversion rates to JE virus among sentinel pigs most directly reflect JE virus activity in the region. Thus, the sero-conversion rate in sentinel pigs, the parameter which is assumed to directly reflect the activity of JE virus, has advantage over the number of JE patients. Furthermore, there have been dramatic changes in rice-growing practices such as mechanization, introduction and withdrawal of insecticides, introduction of growth suppressant and bactericides. These factors also could affect the sero-conversion rates through the effect on the number of mosquitoes.

There have also been reports on the effect of climate factors on vector mosquitoes for JE virus [14,15]. The number of vector mosquitoes is one of the important parameters which affect the epidemic of JE [16]. The present study focused only on the effect of precipitation. We have reported that there were statistically significant positive correlations between sero-conversion rates to JE virus in sentinel pigs, and the daily mean temperatures, the daily maximum and the daily minimum temperatures of the entire year and summertime in the whole country and in the four (north, central, west and south) regions of Japan (Kurane *et al.*, submitted manuscript). There is a possibility that the temperature may be lower when there are higher levels of precipitation. Thus, low temperature during the period with high levels of precipitation may in part contribute to inverse correlation between the levels of precipitation and antibody-positive rate in sentinel pigs. The combined effect of precipitation and temperature is an important subject of research, and needs to be analyzed in the next series of studies.

These results suggest that the relationship between the precipitation and the sero-conversion rate to JE virus is complex. It is possible that not only the levels of precipitation during the designated time period, but also the patterns of rainfall may determine the effects. The effect may be different even with the same levels of precipitation during the designated period between heavy rain for short period of time and low levels of constant rainfall. We could not analyze the rainfall patterns in the present study. Further studies on the effect of the patterns of rainfall and on the combined effects of multiple meteorological parameters are needed.

## 4. Conclusions

We analyzed whether precipitation has any effect on the sero-conversion rates to JE virus in sentinel pigs, a natural host of JE virus. The sero-conversion rate in sentinel pigs best reflects the activity of JE virus in Nature in the countries where JE vaccination has been strongly implemented and most of the population have protective immunity. 

The levels of the annual and summertime precipitation demonstrated statistically significant positive correlations with sero-conversion rates for the whole of the country and for some regions in Japan. On the other hand, the levels of the summertime precipitation demonstrated statistically significant inverse correlations with the sero-conversion rates in other regions. Further, the levels of precipitation during the preceding 10-day periods from days 1–40 before blood sample collection showed inverse correlations with antibody-positive rates in some of the regions. The results indicate that the effect of annual and summertime precipitation on the sero-conversion rate to JE virus is complex; both positive and inverse effects are demonstrated, depending on the regions and possibly the patters of rainfall.
